# Chemical Characterization and Bioactive Properties of Different Extracts from *Fibigia clypeata*, an Unexplored Plant Food

**DOI:** 10.3390/foods9060705

**Published:** 2020-06-01

**Authors:** Gokhan Zengin, Mohamad Fawzi Mahomoodally, Gabriele Rocchetti, Luigi Lucini, Elwira Sieniawska, Łukasz Świątek, Barbara Rajtar, Małgorzata Polz-Dacewicz, Ismail Senkardes, Abdurrahman Aktumsek, Marie Carene Nancy Picot-Allain, Domenico Montesano

**Affiliations:** 1 Department of Biology, Science Faculty, Selcuk University, Campus, 42130 Konya, Turkey; aktumsek@selcuk.edu.tr; 2 Institute of Research and Development, Duy Tan University, Da Nang 550000, Vietnam; mohamadfawzimahomoodally@duytan.edu.vn; 3 Department of Health Sciences, Faculty of Science, University of Mauritius, 80837 Réduit, Mauritius; picotcarene@yahoo.com; 4 Department for Sustainable Food Process, Università Cattolica del Sacro Cuore, Via Emilia Parmense 84, 29122 Piacenza, Italy; gabriele.rocchetti@unicatt.it (G.R.); luigi.lucini@unicatt.it (L.L.); 5 Chair and Department of Pharmacognosy, Medical University of Lublin, 20-093 Lublin, Poland; esieniawska@pharmacognosy.org; 6 Department of Virology, Medical University of Lublin, 20-093 Lublin, Poland; ulubiony.asystent@gmail.com (Ł.Ś.); barbara.rajtar@umlub.pl (B.R.); malgorzata.polz-dacewicz@umlub.pl (M.P.-D.); 7 Department of Pharmaceutical Botany, Pharmacy Faculty, Marmara University, 34854 Istanbul, Turkey; isenkardes@marmara.edu.tr; 8 Department of Pharmaceutical Sciences, Food Science and Nutrition Section, University of Perugia, Via S. Costanzo 1, 06126 Perugia, Italy

**Keywords:** *Fibigia clypeata*, antioxidants, enzyme inhibition, cytotoxicity, mass spectrometry

## Abstract

*Fibigia clypeata* (L.) Medik. is a poorly studied plant species belonging to the Brassicaceae family, and usually used as cress in the salads. The current investigation aimed at assessing the antioxidant potential and inhibitory activity of ethyl acetate, methanol, and aqueous extracts of *F. clypeata* against key enzymes targeted in the management of type II diabetes (α-amylase and α-glucosidase), Alzheimer’s disease (acetylcholinesterase and butyrylcholinesterase), and skin hyperpigmentation (tyrosinase). Cytotoxicity of the extracts was also determined using normal VERO and cancer FaDu and SCC-25 cell lines. Besides, LC-MS was employed to investigate the detailed phytochemical profiles of the extracts. The methanol extract showed potent enzyme inhibitory activity (4.87 mg galantamine equivalent/g, 3.52 mg galantamine equivalent/g, 126.80 mg kojic acid equivalent/g, and 24.68 mg acarbose equivalent/g, for acetylcholinesterase, butyrylcholinesterase, tyrosinase, and α-glucosidase, respectively) and antioxidant potential (96.52, 109.10, 154.02, and 104.85 mg trolox equivalent/g, for DPPH, ABTS, CUPRAC, and FRAP assays, respectively). Interestingly, caffeic acid-*O*-hexoside derivative, caffeyl alcohol *O*-glucopyranoside, and ferulic acid derivative were identified in all extracts. *F. clypeata* extracts showed no cytotoxicity towards VERO cell line and a weak cytotoxic potential against FaDu and SCC-25 cell lines. Interesting scientific evidence gathered from the present study support further investigation on *F. clypeata* in the view of designing and developing a novel therapeutic agent for the management of Alzheimer’s disease, type II diabetes, skin hyperpigmentation problems, as well as cancer.

## 1. Introduction

Ethnopharmacological literature compiled by our ancestors has indicated the potential of plants in the treatment of several health complications affecting mankind. The therapeutic properties of plants have been ascribed to biologically active chemical compounds called secondary metabolites. Alkaloids, terpenoids, and phenolic compounds are the different classes of secondary metabolites, which naturally occur in plants and have been found to exhibit biological effects, such as anti-inflammatory, anti-cancer, anti-diabetic, and antioxidant properties, amongst others [1,2,3,4]. Besides, some of these bioactive compounds (such as polyphenols) possess an important role as dietary constituents, characterized by several health-promoting properties. In the last years, the quest for novel lead candidates for the management of human ailments continues to fuel the study of bioactive compounds from both plants and plant-foods for human nutrition. In particular, several studies have been conducted to test the antioxidant and pharmacological activities of different plant extracts (such as hydroalcoholic, water or ethyl acetate extracts) [5,6,7].

In this direction, the genus Fibigia, belonging to the Brassicaceae family, is distributed in Egypt, Southern Europe, Caucasus, and the Middle East. Brassicaceae, formerly known as Cruciferae because of their 4-petalled flowers, are a very useful edible group of plants. They encompass many edible plants like broccoli, mustard greens, cauliflower, cabbage, kale, collards, turnips, Chinese Bok Choy, and so on. Many important oil-producing plants are in this family. They also tend to have a pungent taste, as in the mustard greens and seeds, and cress. Besides, one cup of a decoction prepared from the leaves and stem of *F. eriocarpa* (DC.) Boiss. is used against the common cold [8]. However, to date, few information exists regarding the use of *F. clypeata* as food ingredient; in fact, to the best of our knowledge, the edible part of this plant (also known as paper pumpkinseed) is the young leaf. In particular, the raw leaves are eaten in the eastern Mediterranean as part of salads. No additional information is provided in the scientific literature regarding other uses as a food ingredient. Overall, a decoction prepared from the stem and fruits of *F. clypeata* (L.) Medik. is used against kidney stones [9], whilst powdered fruits of *F. macrocarpa* Boiss. are used against cattle infertility [10]. *F. clypeata* extracts were previously reported exhibiting anti-leishmanial activities on the intracellular amastigote form of the parasite and induced nitrous oxide production by human macrophages [11]. Therefore, according to the literature, the comprehensive chemical characterization, together with the description of other biological properties (such as enzyme inhibitory and/or anti-cancer potential) of the Fibigia species, is still scarce.

Considering the importance of plant bioactive compounds as related to health-promoting attributes, several recent works analyzed the novel source of phytochemicals by using high-resolution targeted/untargeted mass spectrometry approaches [4,6,7]. In fact, according to the literature [12], using liquid chromatography coupled with mass spectrometry (LC-MS) is recommended to profile and then quantify antioxidant compounds (such as polyphenols) in both plant and food matrices. Therefore, the main goal of this work was to assess the potential enzyme inhibitory activity, in vitro antioxidant properties, and cytotoxicity of the ethyl acetate, methanol, and aqueous extract of *F. clypeata*. Moreover, LC-MS was used to elucidate the detailed phytochemical profile of the different extracts. It is expected that the data generated from this study will provide for the first time valuable and comprehensive scientific information on the poorly studied Fibigia species, serving as the baseline information for future detailed investigations.

## 2. Material and Methods

### 2.1. Plant Material and Preparation of Extracts

The plant material of *F. clypeata* was collected in the area of Hanönü village (Kastamonu, Turkey) in the summer of 2019. Taxonomic identification was performed by the botanist Dr. Ismail Senkardes (Marmara University, Department of Pharmaceutical Botany, Istanbul, Turkey), and 1 voucher specimen was deposited at the herbarium of Selcuk University (MARE-19856). The grinding of naturally dried aerial parts of the plant was carried out by a laboratory mill.

For the extraction step, the maceration technique based on two different organic solvents, namely ethyl acetate (EA) and methanol. For this purpose, samples of the plant material (5 g) were macerated with 100 mL of each solvent for 24 h at room temperature (about 25 °C). Then, the solvents were evaporated under vacuum using a rotary evaporator. The aqueous extract was prepared by traditional infusion technique, and plant material (5 g) was kept with the boiled water (100 mL) for 20 min. Then the water extract was filtered and then lyophilized. All extracts were stored at +4 °C until analysis.

### 2.2. Profiling of Bioactive Compounds in the Different Extracts

To determine total phenolic and flavonoid contents of *F. clypeata* extracts, colorimetric methods were used based on our previous work [13]. In this regard, the results were finally expressed as namely gallic acid equivalents (GAE) for total phenolics and rutin equivalents (RE) for total flavonoids.

Thereafter, the phytochemical analysis of each plant extract was carried out using Agilent 1200 Infinity HPLC and Agilent 6530B QTOF spectrometer (Agilent Technologies, Santa Clara, CA, USA). The conditions of the analyses were described previously [14]. The identification was based on the obtained fragmentation patterns, which were compared to the data available in the scientific literature and the Metlin database (https://metlin.scripps.edu).

### 2.3. Determination of Antioxidant and Enzyme Inhibitory Effects

To detect antioxidant properties, several chemical assays were used, including different mechanisms, namely, radical scavenging, reducing power, and metal chelating. Trolox (TE) and ethylenediaminetetraacetic acid (EDTA) were used as standard antioxidant compounds. Obtained results were expressed as equivalents of these compounds, Grochowski, et al. [15]. To detect inhibitory effects on enzymes, colorimetric enzyme inhibition assays were used, and these assays included tyrosinase, α-glucosidase, α-amylase, and cholinesterases. Some standard inhibitors (galantamine, kojic acid, and acarbose) were used as positive controls.

### 2.4. Cell Assays

#### 2.4.1. Cell Lines and Reagents

In vitro assays were carried out using normal VERO (ECACC, No. 84113001) and cancer FaDu (ATCC, HTB-43) and SCC-25 (ATCC, CRL-1628) cell lines. Cell media used in experiments, antibiotic supplement (Penicillin-Streptomycin Solution), and PBS (phosphate buffer saline) were provided by Corning (Tewksbury, MA, USA), and Foetal Bovine Serum (FBS) by Biowest (Nuaillé, France). The DMF (dimethylformamide) was acquired from Avantor Performance Materials Poland S.A (Gliwice, Poland). The DMSO (dimethyl sulfoxide), MTT (3-(4,5-dimethylthiazol-2-yl)-2,5-diphenyltetrazolium bromide), and hydrocortisone were purchased from Sigma-Aldrich (St. Louis, MO, USA) and SDS (sodium dodecyl sulfate) from AppliChem (AppliChem GmbH, Darmstadt, Germany).

#### 2.4.2. Cell Cultures

The VERO cells were maintained using DMEM (Dulbecco’s Modified Eagle Medium), FaDu cells using Modified Eagle Medium (MEM), whereas SCC-25 required Dulbecco’s Modified Eagle Medium/Nutrient Mixture F-12 (DMEM-F12) supplemented with hydrocortisone. Cell propagation was performed using 10% FBS in appropriate cell media (growth media), whereas the experiments were carried out using media supplemented with 2% FBS. All cell lines were cultivated in media supplemented with Penicillin-Streptomycin Solution and the propagation of cells was carried out at 37 °C in 5% CO_2_ atmosphere (CO_2_ incubator, Panasonic Healthcare Co., Ltd., Tokyo, Japan).

#### 2.4.3. Evaluation of Cytotoxic Properties

The viability of tested cells was assessed using a modified MTT (microculture tetrazolium) test, which measures the ability of succinate dehydrogenase to reduce MTT salt into formazan. Formazan crystals are insoluble in water and are dissolved using a mixture of SDS (14%), DMF (36%), and PBS (50%), producing a purple color. Usually, after overnight incubation, the absorbance was measured at 2 wavelengths—540 and 620 nm. The succinate dehydrogenase activity was correlated with the metabolic activity of the cells allowing to assess the viability of the cells [16]. The stock solutions were obtained by dissolving the samples in DMSO (50 mg/mL) and sterilized using syringe filters (0.2 µm). Trypsinized VERO, FaDu, or SCC-25 cells were suspended in appropriate growth media in the density of 1.5 × 10^5^ cells/mL, 2 × 10^5^ cells/mL or 3 × 10^5^ cells/mL, respectively, and cultured in 96-well plates (Corning). After overnight pre-incubation required for obtaining a semi-confluent cell monolayer, the cells were treated for 72 h with tested extracts in culture media containing 2% FBS in concentrations varying from 1.95 to 1000 µg/mL. Control cells were grown in medium containing 2% of FBS. The cytotoxicity of DMSO used as a sample solvent was also evaluated. After the incubation, all the media was removed. The plates were rinsed with sterile PBS and 100 µL per well of 10% of MTT solution (5 mg/mL) in appropriate media was added. After 4 h of incubation, 100 µL per well of solvent containing sodium dodecyl sulfate (14%), dimethylformamide (36%), and phosphate buffer saline (50%) was added. The next day, the absorbance was measured with the use of an Epoch reader (BioTek Instruments, Inc., Winooski, VT, USA) running Gen5 program (2.01.14; BioTek) and data were exported to the GraphPad Prism (v8.0.1) for further analysis. The viability of cells treated with tested samples was compared with control cells, and the values of CC_50_ (concentration resulting in 50% inhibition) and CC_10_ (concentration resulting in 10% inhibition) were estimated based on dose-response curves.

### 2.5. Statistical Analysis

The analysis of the variance (one-way ANOVA) followed by Duncan’s Multiple Range post hoc test (*p* < 0.05) was done using the data from in vitro spectrophotometric and enzymatic assays. In particular, the software PASW Statistics 26.0 (SPSS Inc., Chicago, IL, USA) was used. Therefore, means without a common superscript letter showed significant differences (*p* < 0.05). Pearson’s correlations (*p* < 0.05; two-tailed) were then calculated using PASW Statistics 26.0. Regarding the cell assays, the tests were carried out in triplicate and rerun at least thrice. For statistical evaluation, a 2-way ANOVA followed by Tukey’s multiple comparison test was used.

## 3. Results and Discussion

### 3.1. Phytochemical Profile Determination

In this work, standard in vitro spectrophotometric analyses was used to determine the total phenolic and flavonoid contents of the ethyl acetate, methanolic, and aqueous extracts of *F. clypeata*. The results are presented in Table 1.

**Figure 1:**
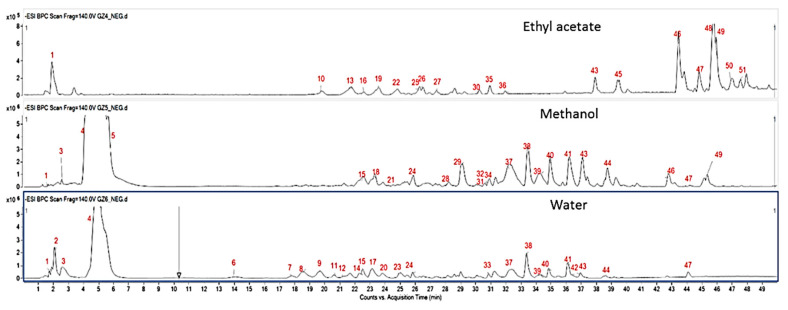
Chromatograms of the different *F. clypeata* extracts (i.e., ethyl acetate, methanol, and water), as result by HPLC-ESI-QTOF analysis. The numbers of compounds (red color) correspond to those reported in Table 2.

As can be observed from the table, the highest phenolic (33.42 mg GAE/g) and flavonoid (21.58 mg RE/g) contents were obtained when considering the methanolic extract. Besides, a strong correlation coefficient (*p* < 0.01) of 0.809 was found between the two different assays (Supplementary Material Table S1). In our previous study on *F. eriocarpa* [16], the highest total phenolic level was determined in ethyl acetate extract (41.87 mg GAE/g), followed methanol (35.42 mg GAE/g) and water (33.63 mg GAE/g). Similar to current results, methanol extract (24 mg RE/g) of *F. eriocarpa* contained the highest level of flavonoids. In accordance with our results, several researchers reported that methanol was one of the best solvents to extract flavonoids [17,18,19]. However, spectrophotometric analyses are mainly able to provide an insight into the content of the bioactive compound of herbal extracts without providing detailed phytochemical composition. Besides, a possible interference of non-phenolic compounds characterizing the extracts has been reported yielding false-positive results [12]. Therefore, in the present study, high performance liquid chromatography coupled to mass spectrometry was used to assess the detailed profile of *F. clypeata* extracts. The identification of compounds was performed by HPLC-ESI-QTOF mass spectrometry in negative ionization mode. The tentatively identified compounds from the extracts were summarized in Table 2, and chromatograms were reported in Figure 1.

Overall, in our experimental conditions, **51** compounds were tentatively identified, mainly consisting of polyphenols and phenolic derivatives, such as flavonoids and phenolic acids. Interestingly, we also found two glucosinolates (i.e., compounds **4** and **5**), namely *p*-Methoxy-2-hydroxy-2-phenylethyl glucosinolate and *p*-Methoxy-2-hydroxy-2-phenylethyl glucosinolate—desulfo, previously described in crucifer seeds [22]. In addition, **1**, **43**, and **57**, corresponding to a caffeic acid-*O*-hexoside derivative, a caffeyl alcohol *O*-glucopyranoside, and a ferulic acid derivative, respectively. These latter were tentatively identified in all the extracts tested. In addition, compounds **6** and **7**, **8** and **9**, exhibited the same fragmentation patterns and were characterized from the water extract as dihydroxybenzoic acid hexosides and *p*-coumaric acid ethyl ester derivatives. The fragmentation of compound **16** corresponded to *p*-coumaric acid hexoside, which was tentatively identified from the ethyl acetate extract. Compound **31**, with [M-H]- at *m*/*z* 193, was identified as ferulic acid. Moreover, several sesquiterpenes (i.e., compounds **44**, **46**, and **51**) were also tentatively characterized (Table 2).

### 3.2. Enzyme Inhibition Activity

Enzyme inhibitors hold a significant share in clinically approved drugs, and their attractiveness has been associated with their specific roles in several metabolic pathways. Drug discovery and development focus on identifying and optimizing lead compounds that act on specific enzymes [26]. In the present study, the ability of *F. clypeata* extracts to inhibit key enzymes targeted in the management of Alzheimer’s disease, skin hyperpigmentation, and type II diabetes was assessed. About 50 million people worldwide have dementia and Alzheimer’s disease, the most common form of dementia accounts for 60–70% of the cases [27]. Based on the cholinergic hypothesis, the lack of neurotransmitters hinders connections between neurons and thus affects the brain’s neuronal circuits [28]. The inhibition of acetylcholinesterase has been advocated in the management of mild cognitive impairment due to Alzheimer’s disease. Clinically approved drugs, such as donepezil, galantamine, and rivastigmine, containing acetylcholinesterase inhibitor as an active ingredient, are currently used in the management of Alzheimer’s disease [29]. Later, the function of another cholinesterase enzyme, namely, butyrylcholinesterase, has also been evoked. The increased activity of butyrylcholinesterase, up to 120%, in the late stage of Alzheimer’s disease has been related to the wasting away of the brain and aggravation of behavioral and cognitive dysfunction in Alzheimer’s disease patients [30]. Besides, it has been reported that butyrylcholinesterase could compensate a lack of acetylcholinesterase in the acetylcholinesterase knockout mice model [31]. These facts have brought the role and need for butyrylcholinesterase inhibition into the limelight. Finding a novel candidate showing both acetylcholinesterase and butyrylcholinesterase inhibitory activity represents an interesting therapeutic strategy for the management of Alzheimer’s disease. However, as reported in the literature, further ad-hoc studies are strongly required to find also possible markers of the neurodegenerative disease [32]. As can be observed from Table 3, the ethyl acetate and methanol extracts of *F. clypeata* showed inhibitory action against both cholinesterase enzymes.

The methanolic extract (4.87 mg GALAE/g) was most active against acetylcholinesterase. Both the ethyl acetate (3.54 mg GALAE/g) and methanol (3.52 mg GALAE/g) extracts showed comparable inhibition against butyrylcholinesterase. In our previous study, the best cholinesterase inhibition abilities of *F. eriocarpa* were reported for ethyl acetate (2.12 mg GALAE/g for AChE and 2.01 mg GAELAE/g for BChE) and methanol (1.83 mg GALAE/g for AChE and 1.08 mg GALAE/g for BChE) extracts [16]. Based on these values, the ethyl acetate and methanol extracts of *F. clypeata* were stronger than *F. eriocarpa*. In the literature survey, several researchers reported the significant cholinesterase inhibitory abilities for other solvents (ethyl acetate, chloroform, and methanol, etc.) rather than water [33,34,35]. This fact could be explained to extract low polarity compounds such as alkaloids and terpenoids with these solvents, and they could be more active on cholinesterases.

Likewise, the methanolic and ethyl acetate extracts of *F. clypeata* showed potent inhibition against tyrosinase. The inhibition of tyrosinase is crucial in the management of skin hyperpigmentation conditions, such as melasma and freckles. Inhibiting tyrosinase is directly related to the reduced production of the dark brown melanin. Moreover, the increased public interest towards naturally derived products, including cosmetic and dermatological products, has fueled the need for natural tyrosinase inhibitors. Another species of the *Fibigia* genus, namely *F. eriocarpa*, was previously reported to inhibit tyrosinase [16]. In contrast to our present findings, the ethyl acetate and methanol extracts of *F. eriocarpa* were not active on tyrosinase. This fact could be explained with the differences of chemical profiles in these extracts and the complex interactions of phytochemicals. However, methanolic extracts exhibited stronger actions on tyrosinase in earlier studies conducted by some researchers [36,37].

Monitoring hyperglycemia, the hallmark of type II diabetes, is playing a pivotal role in the management of the disease. Apart from dietary modifications, the inhibition of carbohydrate hydrolyzing enzymes assists in maintaining a normal glycemic level. The inhibition of α-amylase, which is situated in the upper gastrointestinal tract and catalyzes the initial breakdown of ingested polysaccharides into oligosaccharides and the inhibition of α-glucosidase, which is situated in the brush border of the small intestine and catalyzes the last step of carbohydrate digestion, significantly prevents glycemic peaks. However, prominent α-amylase inhibition has been associated with gastrointestinal discomforts caused by the fermentation of undigested carbohydrates in the colon. In the present study, a low α-amylase inhibition was observed while the ethyl acetate (22.32 mmol ACAE/g) and methanol (24.68 mmol ACAE/g) extracts of *F. clypeata* exhibited potent α-glucosidase inhibitory action. It was noted that, in general, the water extract of *F. clypeata* exhibited the lowest enzymatic inhibition. The higher activity of the ethyl acetate and methanol extracts might be related to the synergistic action of the different bioactive compounds present in these extracts. Anti-diabetic properties of the ethyl acetate and methanol extracts of *F. clypeata* were stronger than those of *F. eriocarpa* (amylase: 0.44 mmol ACAE/g for ethyl acetate and 0.43 mmol ACAE/g for methanol; glucosidase: 5.01 mmol ACAE/g for ethyl acetate and 1.57 mmol ACAE/g for methanol) in our previous paper. Additionally, it is worth mentioning the level of the different bioactive compounds might have also affected enzyme activity. The alkaloidal amine, sinapine, tentatively characterized from the ethyl acetate extract of *F. clypeata* was previously reported to inhibit acetylcholinesterase isolated from rat cerebral homogenate with an IC50 of 3.66 µmol/L [38]. Regarding correlations between total phenolic-flavonoid content with enzymatic assays, we found strong correlation coefficients when considering only TFC (total flavonoid content) values. In this regard, total flavonoids were strongly correlated to AChE (0.870; *p* < 0.01), tyrosinase (0.795; *p* < 0.05), α-glucosidase (0.771; *p* < 0.05), and BChE (0.705; *p* < 0.05) inhibition values (Table S1).

### 3.3. In Vitro Antioxidant Activity of the Tested Extracts

The role of oxidative stress in the onset and/or progression of human ailments supports the systematic antioxidant evaluation of studied plant extracts. Antioxidants can act by different mechanisms, namely, hydrogen atom transfer, single electron transfer, or transition metal chelation [39]. In this study, multiple antioxidant assays were used to obtain a comprehensive understanding of the antioxidant properties of the *F. clypeata* extracts. As presented in Table 4, the methanolic extract (96.52 and 109.10 mg TE/g, for DPPH and ABTS, respectively) of *F. clypeata* showed potent radical scavenging properties.

Likewise, the methanolic extract exhibited the highest reducing potent in the CUPRAC (154.02 mg TE/g) and FRAP (104.85 mg TE/g) assays. Obtained results could be compared with earlier studies on other *Fibigia* or Brassicaceae species. For example, the antioxidant properties in *F. eriocarpa* extracts can be ranked methanol > ethyl acetate > water. However, the antioxidant abilities of the water extract of *F. clypeata* were stronger than that of *F. eriocarpa*. [16] Besides, different levels for antioxidant properties in some Brassicaceae species have been reported in the literature [40,41,42]. At this point, the chemical profiles of the extracts were closely related to their antioxidant properties. In this sense, several compounds present in *F. clypeata* methanolic extract might be responsible for the observed activity. Sinapic acid was reported to exhibit radical scavenging potential [43]. Caffeic acid has been reported to be an effective radical scavenger and reducing agent as well as a metal chelator [44]. In terms of metal chelating, the aqueous extract of *F. clypeata* showed the highest activity. These findings suggest that bioactive compounds present in the methanolic extract exhibited mostly radical scavenging and reducing potential, while compounds present in the aqueous extract were potential metal chelators. Regarding the potential correlation between TPC and TFC values, we found strong correlation coefficients between TPC and some antioxidant assays, such as DPPH (0.943; *p* < 0.01), CUPRAC (0.954; *p* < 0.01), FRAP (0.874; *p* < 0.01), and ABTS (0.790; *p* < 0.05) values. In addition, total flavonoids were inversely correlated to chelating activity (−0.902; *p* < 0.01) and strongly correlated to DPPH values (0.876; *p* < 0.01) (Table S1).

### 3.4. Cell Assays

To assess the anti-cancer potential of *Fibigia clypeata* extracts, their cytotoxicity was measured on two cancer cell lines belonging to the group of head and neck squamous cell carcinomas (HNSCC), i.e., squamous cell carcinoma of the pharynx (FaDu) and squamous cell carcinoma of the tongue (SCC-25) and compared with the results obtained for the normal VERO cell line. The results of cytotoxicity evaluation are presented in Table 5.

**Figure 2:**
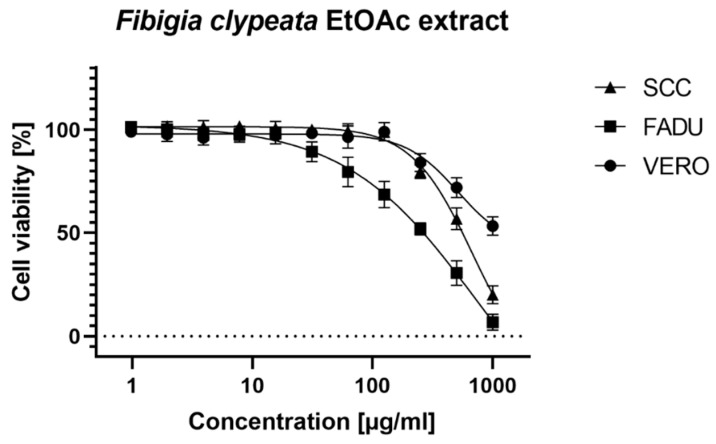
Cytotoxicity of *Fibigia clypeata* ethyl acetate extract.

In the case of VERO cells, no cytotoxicity was observed for methanolic and aqueous extracts. The ethyl acetate extract showed a cytotoxic effect in concentrations above 125 µg/mL, but it was impossible to calculate the CC50 value because even in the highest tested concentration of 1000 µg/mL the viability of VERO cells was above 50% (Figure 2).

**Figure 3:**
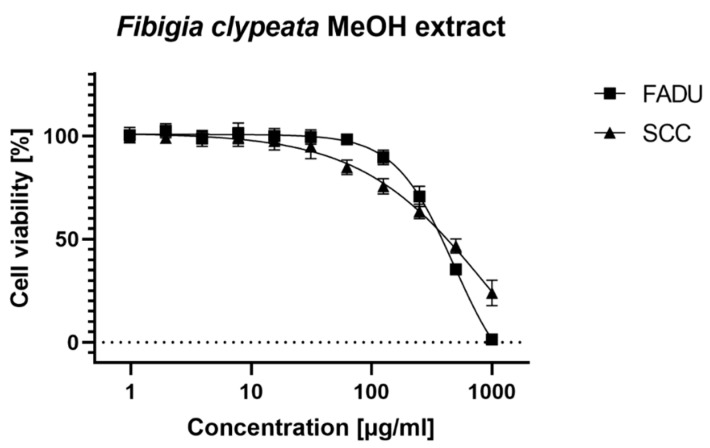
Cytotoxicity of Fibigia clypeata methanolic extract.

**Figure 4:**
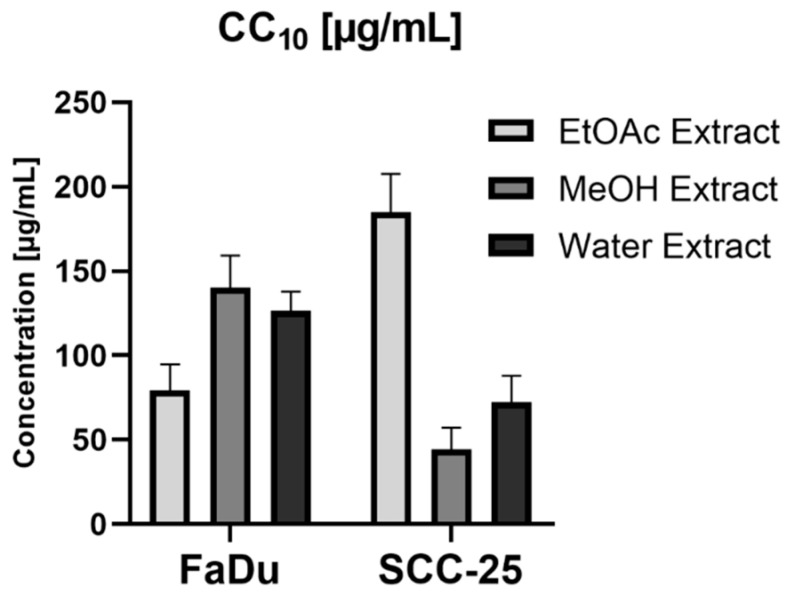
The CC10 values of Fibigia clypeata extracts on cancer cell lines.

Similarly, it was impossible to calculate CC50 for aqueous extract on FaDu cells. Both ethyl acetate and methanolic extracts showed selective toxicity towards FaDu cells. In the case of SCC-25 cell line, selective toxicity was observed for all tested extracts. The CC50 and CC10 values of ethyl acetate extract on both cancer cell lines showed a statistically important difference (*p* < 0.001). In the case of methanolic extract tested on FaDu and SCC-25, the CC50 values were similar, but a significant difference was found for the CC10 values (*p* < 0.001) (Figure 3 and Figure 4).

The criteria of classification of plant extract cytotoxicity according to the protocols suggested by the National Cancer Institute (NCI) [45] and selected literature are shown in Table 6 [45,46,47,48].

According to this criterion, all tested extracts can be classified as non-cytotoxic towards the VERO cell line. In the case of cancer cell lines, ethyl acetate and methanolic extracts tested on FaDu and methanolic and aqueous extracts tested on SCC-25 can be classified as weakly cytotoxic. However, the selective activity towards cancer cell lines encourages further investigations focused on the isolation of bioactive constituents and testing their anti-cancer potential. Di Giorgio et al. [11] studied the immunomodulatory and anti-leishmanial activities of Lebanese plants and reported the cytotoxicity of *F. clypeata* extracts assessed on the THP1 human monocyte cell line by colorimetric determination of cell viability using the oxidation–reduction indicator Alamar Blue. The aqueous and dichloromethane extracts showed CC50 of 124.9 and 123.6 µg/mL, whereas in the case of methanol extract, it was above 250 µg/mL.

## 4. Conclusions

The present work presents interesting scientific data on the potential of *Fibigia clypeata* in the management of Alzheimer’s disease, type II diabetes, and skin hyperpigmentation problems. Cytotoxicity studies also supported the action of *F. clypeata* against cancerous cell lines, thereby advocating further investigation geared towards assessing the anti-cancer properties of *F. clypeata*. Higher enzyme inhibition and in vitro antioxidant capabilities were noted for the methanolic extract, showing that the choice of solvent greatly affected the extraction of bioactive compounds from the plant material. Therefore, considering the potential health-implication given by this plant-food for human nutrition, both in vivo and bioavailability studies appear to be strongly required to further support the preliminary data collected in the present study.

## Figures and Tables

**Table 1:** Total bioactive components in the tested extracts. Values are expressed as the mean (*n* = 3) ± standard deviation. GAE: Gallic acid equivalent; RE: Rutin equivalent. Different superscript letters in the same column indicate significant differences (*p* < 0.05), as determined by Duncan’s post-hoc test.

Extracts	Total Phenolic Content (mg GAE/g)	Total Flavonoid Content (mg RE/g)
Ethyl acetate	21.04 ± 0.68 ^a^	10.13 ± 0.39 ^b^
Methanolic	33.42 ± 3.79 ^b^	21.58 ± 0.16 ^c^
Water	24.43 ± 0.21 ^a^	5.81 ± 0.07 ^a^

**Table 2:** Tentative identification of phytochemical compounds characterizing the different extracts, as resulted by HPLC-ESI-QTOF-MS analysis.

Compound	Tentative Identification	RT (Min)	Molecular Formula	Molecular Weight	[M-H]	Fragments (*m/z*)	Extracts	References
**1**	Caffeic acid-*O*-hexoside derivative	1.725		388.1247	387.1247	341.1180; 179.0121	EA, MeOH, Water	[20]
**2**	Ethyl 2-hydroxy-3-(hydroxyphenyl)propanoate	2.120	C_11_H_14_O_4_	210.0051	209.0051	190.9851; 172.9663; 146.9663; 128.9377; 102.9125	Water	[21]
**3**	Quinic acid	2.773	C_7_H_12_O_6_	192.0205	191.0205	173.0034; 127.0437; 111.0463	MeOH, Water	[20]
**4**	*p*-Methoxy-2-hydroxy-2-phenylethyl glucosinolate	4.825	C_16_H_23_NO_11_S_2_	469.0042	468.0042	388.0458; 274.9727; 259.0033; 240.9840; 225.9687; 194.9916; 139.3081; 135.9009	MeOH, Water	[22]
**5**	*p*-Methoxy-2-hydroxy-2-phenylethyl glucosinolate–desulfo	5.018	C_16_H_23_NO_8_S	389.0527	388.0455	274.9727; 259.0033; 240.9840; 225.9687; 194.9916; 139.3081; 135.9009	MeOH, Water	[22]
**6**	Dihydroxybenzoic acid hexoside	15.386	C_13_H_16_O_9_	316.0741	315.0741	153.0440; 109.0311	Water	[20]
**7**	Dihydroxybenzoic acid hexoside	17.781	C_13_H_16_O_9_	316.0772	315.0772	153.0440; 109.0311	Water	[20]
**8**	*p*-Coumaric acid ethyl ester derivative	18.460		314.0613	313.0613	191.0031; 172.9730; 1146.9803; 128.9525	Water	[20]
**9**	*p*-Coumaric acid ethyl ester derivative	19.622		314.0613	313.0613	191.9672; 172.9696; 146.9676; 128.9426	Water	[20]
**10**	2-hydroxy-3-phenylpropanoic acid hexoside	20.062	C_15_H_20_O_8_	328.1154	327.1154	165.0021; 146.9606; 128.9594; 100.9476	EA	[23]
**11**	Rhamnoside of ethyl 2-hydroxy-3-(hydroxyphenyl)propanoate isomer	20.466	C_17_H_24_O_8_	356.0780	355.0780	209.0037; 191.0097; 181.3211; 163.0022; 146.9911	Water	[23]
**12**	Rhamnoside of ethyl 2-hydroxy-3-(hydroxyphenyl)propanoate isomer	21.598	C_17_H_24_O_8_	356.0780	355.0780	209.0401; 191.0162; 181.3317; 163.0043; 146.9924	Water	[23]
**13**	Benzoic acid	21.641	C_7_H_6_O_2_	122.0369	121.0369	-	EA	[23]
**14**	Feruloyl, ethyl 2-hydroxy-3-(hydroxyphenyl)propanoate	22.395	C_17_H_22_O_10_	386.0912	385.0912	209.0082; 190.9877; 146.9696; 129.9443	Water	[23]
**15**	Sinapic acid hexoside derivative	22.488		432.2047	431.2047	385.2424; 223.1204	MeOH, Water	[24,25]
**16**	*p*-Coumaric acid hexoside	22.687	C_15_H_18_O_8_	326.0987	325.0987	162.9889.;144.9626; 119.0003	EA	[20]
**17**	*p*-Coumaric acid ethyl ester derivative	23.203		314.0613	313.0613	190.9426; 172.9625; 146.9600; 128.9344; 120.9409	Water	[20]
**18**	*p*-coumaroyl acetic acid derivative	23.407		388.1763	387.1763	207.0850; 163.0775	MeOH	[21]
**19**	Protocatechuic acid derivative	23.613		432.2013	431.2013	385.1909; 223.1168; 153.0360	EA	[21]
**20**	Rhamnoside of ethyl 2-hydroxy-3-(hydroxyphenyl)propanoate	23.976	C_17_H_24_O_8_	356.0780	355.0780	209.0074; 190.9903	Water	[23]
**21**	(Epi)catechin	24.569	C_15_H_14_O_6_	290.0440	289.0440	245.0665; 205.0202; 125.9405	MeOH	[20]
**22**	Phenylacetic acid	24.773	C_8_H_8_O_2_	136.0057	135.0057	-	EA	[20]
**23**	Feruloyl, ethyl 2-hydroxy-3-(hydroxyphenyl)propanoate	24.994	C_17_H_22_O_10_	386.0912	385.0912	223.01146; 209.0082; 190.9877; 146.9696; 129.9443	Water	[23]
**24**	*O*-sinapoyl-glucose	25.870	C_17_H_22_O_10_	386.1268	385.1268	223.0670;	MeOH, Water	[25]
**25**	Vanilic acid derivative hexoside	26.244		508.2029	507.2029	345.1089; 327.1215; 315.1409; 167.0116	EA	[21]
**26**	Ellagic acid	26.397	C_14_H_6_O_8_	302.1221	301.1221	283.1089; 271.1000; 160.9988; 125.9795	EA	[21]
**27**	Sinapic acid derivative	27.423		312.1533	311.1533	223.8222; 208.0325	EA	[24,25]
**28**	Caffeic acid derivative	28.138		416.2092	415.2092	179.0079; 137.0253	MeOH	[21]
**29**	Quercetin-*O*-hexoside	29.159	C_21_H_20_O_12_	464.1014	463.1014	301.0300; 178.9275; 150.9379	MeOH	[20]
**30**	Dimethoxy-trihydroxyflavone	30.177	C_17_H_14_O_7_	300.1051	299.1051	269.0865	EA	[23]
**31**	Ferulic acid	30.672	C_10_H_10_O_4_	194.0153	193.0153	160.9938; 151.0017; 133.9934	MeOH	[21,23]
**32**	Kaempferol glucoside	30.710	C_21_H_20_O_11_	448.1060	447.1060	285.0817	MeOH	[14]
**33**	Caffeic acid derivative	30.788		362.1790	361.1790	193.0563; 179.0297; 165.0047; 146.9725; 135.9938; 120.9468	Water	[20]
**34**	Quercetin derivative	30.905		550.0995	549.0995	505.1463; 301.0477	MeOH	[20]
**35**	Gallic acid derivative	31.085		188.0603	187.0603	169.0445; 125.0163	EA	[21]
**36**	Caffeic acid derivative	32.040		362.1765	361.1765	301.1126; 285.2249; 179.0294; 165.0075; 146.9873; 120.9487	EA	[21]
**37**	Unidentified	32.203		306.0734	305.0734	225.0952	MeOH, Water	
**38**	Unidentified dimer	33.506		896.3362	895.3362	447.1766; 403.1865; 343.1615	MeOH, Water	
**39**	Dihydrocoumaric acid derivative	34.565		246.0071	244.9989	165.0111	MeOH, Water	[20]
**40**	Trihydroxy-methoxyflavone derivative hexoside	35.032		920.2537	919.2537	757.2071; 461.1288; 299.0536; 284.0217	Water	[14]
**41**	Unidentified	36.106		534.1841	533.1841	445.2067; 385.1792; 343.1590; 163.0220	Water	
**42**	Tetrahydroxy-dimethoxyflavone	36.744	C_17_H_14_O_8_	346.0740	345.0740	330.0496; 315.0293	Water	[14]
**43**	Caffeyl alcohol O-glucopyranoside	37.102	C_15_H_20_O_8_	328.2227	327.2227	229.1231; 165.0727; 121.0198	EA, MeOH, Water	[23]
**44**	Sesquiterpene-derivative	38.691		330.2386	329.2386	289.1532; 229.1252; 211.1104; 129.0162	MeOH, Water	
**45**	Trihydroxy-methoxyflavone	39.570	C_16_H_12_O_6_	300.0572	299.0572	284.0313	EA	[14]
**46**	Sesquiterpene-derivative	43.427		308.1944	307.1944	289.1829; 235.1171; 209.0913; 185.0827; 120.9816	MeOH, Water	
**47**	Ferulic acid derivative	44.078		294.1770	293.1770	236.0898; 221.1337; 193.1262	EA, MeOH, Water	[21]
**48**	Phenylacetic acid derivative	45.795		306.1786	305.1786	249.1359; 135.0091; 125.0171;	EA	[20]
**49**	Sinapic acid derivative	45.927		312.2265	311.2265	223.1479; 208.4343	MeOH, Water	[24,25]
**50**	Sinapine	46.770	C_16_H_24_NO_5_	310.2099	309.2099	291.2122; 251.1664; 223.1588; 208.6589	EA	[25]
**51**	Sesquiterpene-derivative	47.632		310.2099	309.2099	291.1943; 225.1275; 197.0855; 110.9822	EA	

**Table 3:** Enzyme inhibitory activities of the tested extracts. Values are expressed as mean (*n* = 3) ± standard deviation. GALAE: Galantamine equivalent; KAE: Kojic acid equivalent; ACAE: Acarbose equivalent; na = not active. Different superscript letters in the same column indicate significant differences (*p* < 0.05), as determined by Duncan’s post-hoc test.

Extracts	AChE Inhibition (mg GALAE/g)	BChE Inhibition (mg GALAE/g)	Tyrosinase Inhibition (mg KAE/g)	Amylase Inhibition (mmol ACAE/g)	Glucosidase Inhibition (mmol ACAE/g)
Ethyl acetate	3.49 ± 0.16 ^b^	3.54 ± 0.43 ^b^	115.07 ± 3.14 ^b^	0.73 ± 0.04 ^c^	22.32 ± 1.15 ^b^
Methanolic	4.87 ± 0.57 ^c^	3.52 ± 0.11 ^b^	126.80 ± 0.39 ^c^	0.55 ± 0.03 ^b^	24.68 ± 0.03 ^c^
Water	na ^a^	na ^a^	43.11 ± 5.14 ^a^	0.11 ± 0.01 ^a^	1.06 ± 0.20 ^a^

**Table 4:** In vitro antioxidant properties of the tested extracts. Values are expressed as the mean (*n* = 3) ± standard deviation. TE: Trolox equivalents; EDTAE: EDTA equivalents. Different superscript letters in the same column indicate significant differences (*p* < 0.05), as determined by Duncan’s post-hoc test.

Extracts	DPPH (mg TE/g)	ABTS (mg TE/g)	CUPRAC (mg TE/g)	FRAP (mg TE/g)	Phosphomolybdenum (mmol TE/g)	Chelating Ability (mg EDTAE/g)
Ethyl acetate	22.38 ± 0.37 ^a^	15.61 ± 0.58 ^a^	78.41 ± 4.04 ^a^	28.53 ± 1.47 ^a^	1.91 ± 0.05 ^b^	25.21 ± 1.20 ^b^
Methanolic	96.52 ± 0.10 ^c^	109.10 ± 2.57 ^c^	154.02 ± 4.58 ^c^	104.85 ± 0.57 ^c^	1.79 ± 0.15 ^b^	21.18 ± 1.72 ^a^
Water	40.21 ± 1.24 ^b^	90.98 ± 1.00 ^b^	108.02 ± 1.01 ^b^	75.18 ± 0.77 ^b^	1.25 ± 0.11 ^a^	31.64 ± 0.87 ^c^

**Table 5:** Cytotoxicity potential of the tested extracts. na = not applicable.

	CC_50_ (µg/mL)		
	Ethyl Acetate	Methanol	Water
VERO	>500	na	na
FaDu	231.6 ± 13.97	363.13 ± 19.56	>500
SCC-25	554.57 ± 62.34	408.43 ± 48.52	412.43 ± 51.61

**Table 6:** Classification of plant extracts cytotoxicity according to National Cancer Institute.

CC_50_ Value	<20 μg/mL	21–200 μg/mL	201–500 μg/mL	>500 μg/mL
Cytotoxic activity	high	moderate	weak	no activity

## Supplementary Materials

The following are available online at https://www.mdpi.com/2304-8158/9/6/705/s1, Table S1: Pearson’s correlation coefficients considering the different in vitro spectrophotometric and enzymatic assays.
